# Perceived Patient Satisfaction and Associated Factors among Psychiatric Patients Who Attend Their Treatment at Outpatient Psychiatry Clinic, Jimma University Medical Center, Southwest Ethiopia, Jimma, 2019

**DOI:** 10.1155/2020/6153234

**Published:** 2020-03-04

**Authors:** Chalachew Kassaw, Elias Tesfaye, Shimelis Girma, Liyew Agenagnew

**Affiliations:** ^1^Department of Psychiatry, College of Health Science, Dilla University, P.O. Box 419, Dilla, Ethiopia; ^2^Department of Psychiatry, College of Health Science, Jimma University, P.O. Box 378, Jimma, Ethiopia

## Abstract

**Background:**

In health care, patient satisfaction is an attitudinal response and a pillar for quality assurance, but there is reluctance to measure it among mentally ill patients. Satisfied patients become more compliant. However, no study was done in this study area before. Therefore, this study was conducted to determine the magnitude of perceived patient satisfaction and associated factor at Jimma University Medical Center, outpatient psychiatry clinic.

**Methods:**

Cross-sectional study design was conducted, and systematic random sampling technique was used to get study participants. The 24-item Mental Health Service Satisfaction Scale (a validated tool in Ethiopia) was used to assess patient satisfaction. Data was entered using Epi-data 3.1 and exported to the Statistical Package for the Social Sciences 22.0 for analysis. Linear regression analysis (*P* < 0.05) was used to identify the association between the outcome and independent variable.

**Result:**

414 respondents participated in the study with response rate of 98%. The overall percentage of patient satisfaction was 50.3% (95% CI 48.4%–51.2%). Being male (*β* = −0.651, 95% CI (-0.969, -0.332)), having secondary and above educational status (*β* = −0.651, 95% CI (-0.969, -0.332)), having secondary and above educational status (*β* = −0.651, 95% CI (-0.969, -0.332)), having secondary and above educational status (*β* = −0.651, 95% CI (-0.969, -0.332)), having secondary and above educational status (*β* = −0.651, 95% CI (-0.969, -0.332)), having secondary and above educational status (*β* = −0.651, 95% CI (-0.969, -0.332)), having secondary and above educational status (*β* = −0.651, 95% CI (-0.969, -0.332)), having secondary and above educational status (*β* = −0.651, 95% CI (-0.969, -0.332)), having secondary and above educational status (*β* = −0.651, 95% CI (-0.969, -0.332)), having secondary and above educational status (*Conclusion and Recommendation*. This study found that half of the study participants are satisfied with the service. Distance from the hospital, current substance use, waiting time, and having good social support were identified as modifiable factors that can be improved through working with stakeholders to increase patient satisfaction.

## 1. Introduction

Satisfaction is something that fulfills expectation and desire and giving what is required [1]. In health care, satisfaction is multidimensional, which is not tightly defined, and in addition, it is an attitudinal response that is very subjective, cognitively based, and emotionally affected [2].

The Donabedian theory of quality of health care plays a basis for research to be done in the area of quality assurance through involving service users [3].

Over the past few decades, patients' opinions about their treatment have been getting attention and are being considered as the measure of quality health indicator, which is associated with compliance and health outcome [4, 5].

Across the United States of America and Europe, measuring tools were not universally defined; also in low- and middle-income countries, measuring tools were positively framed; and patients were chosen agree or disagree for answer, so that positive responses may reflect either true satisfaction or bias induced by the positive framing [6, 7].

There was a reluctance to measure the level of patient satisfaction among mentally ill patients about their treatment through time because of a debate of whether they can give valid comments on their treatment or not; but through time, the development of questionnaires that claim to “reliably measure” the views of patients has coincided with a greater acceptance for study on patient satisfaction [8].

In Ethiopia, out of 53 outpatient mental health services, half of the outpatient facilities have at least one psychotropic medicine of each medication group, and about 114.79 per 100000 populations were visiting an outpatient mental health service [9, 10].

Even if health care satisfaction is multidimensional, since health is a human right, WHO advocates health institutions to give more emphasis on client-centered services to become more responsive to user need and to respond in a timely manner to improve the quality of care [11, 12]. The global patient satisfaction in all types of illness was 66%, which ranges from 72% in developed countries to 60% in developing countries [13]; and outpatient mental health service in Europe was from 90% [14] to 45% [15], in Africa from 72% [16] to 45% [15], and in Ethiopia from 77% [17] to 57% [18].

Factors that were affecting patient satisfaction in mental health services were sociodemographic-related factors, clinical-related factors, social factors, and service-related factors [19–21].

Health institutions that periodically performed service patient satisfaction level showed high-quality assurance and efficiency of care through decreasing referrals and readmissions, and their patients were more compliant, cooperative, and interested to be involved actively in their treatment regimen; promptness of follow-up and continuity of outpatient care; good treatment response, and in addition, they addressed reliability of services through providing services in a consistent and dependable manner and decrease burnout of the health-care providers [22–27].

In European countries, various measures were taken to increase the patient satisfaction; among that, the most common were giving training for physicians about participatory decision-making styles, experiential relationship-centered physician communication skills, psychoeducation about treatment, improved community services, staff training, and implementation of standard policies and guidelines; and in low- and middle-level countries, even if there was no sufficient evidence for researches done, they use post a record of cleaning activity in toilets and in patient wards, distribute leaflets in the local language with each prescription, and share ideas about patient experience across the hospital to increase patient satisfaction [28–30].

Even if patient satisfaction is a reliable predictor of quality health care, initially, there was reluctance to measure mental health service satisfaction by the patients. So this study will be important at first to break the reluctance history of measuring mental health service satisfaction by the patient. Second, the result from this study will determine the current magnitude and associated factors of patient satisfaction, which will be vital for intervention purposes. Third, this study result will be important for patients to increase their level of confidence to decide to be involved in their treatment; and also, this is important for staff in identifying and working on those identified factors that hinder patient satisfaction. Finally, this study result will be crucial for policy makers, hospital administrators, and nongovernmental organizations to design locally relevant and sustainable interventional policy that helps to increase patient satisfaction and finally to achieve quality mental health care.

Moreover, since patient need and attitude towards service change from time to time, this study result will be important for researchers to use as baseline information for future assessment of this study area and also for other study areas working on patient satisfaction.

## 2. Methods and Materials

### 2.1. Study Area and Period

This study was conducted from April 12 to May 12, 2019, at Jimma University Medical Center (JUMC) outpatient psychiatry clinic, which is located in southwest Ethiopia, 352 km from Addis Ababa [26].

### 2.2. Study Design

An institutional-based cross-sectional study design was employed.

### 2.3. Eligibility Criteria

#### 2.3.1. Inclusion

All patients age 18 and above who attended for at least 6 months at an out-patient psychiatry service were included.

#### 2.3.2. Exclusion Criterion

Patients who were not able to respond due to different disabilities to the interviewer question for the study were excluded.

### 2.4. Sample Size Calculation

It was calculated by using a single proportion formula, but since the outcome variable was continuous, to calculate the sample size, standard deviation was used, where *n* is the required sample size

(1) $$\begin{array}{c} n={\left(\frac{Z\alpha }{2}\right)}^{2}\frac{\sigma 2}{d^{2}}=\frac{\left(1.96\right)\;\left(1.96\right)\;\left(0.5\right)\;\left(0.5\right)}{\left(0.05\right)\;\left(0.05\right)}=384, \end{array}$$

where *σ* is for the unknown variance (0.5), *Z* is the reliability coefficient at 95% confidence interval (1.96), *W* is the margin of error (0.05), and *N* is the nonresponse rate (10%).

The total sample size was 384 + 38.4 = 422.

### 2.5. Sampling Procedure

A systematic random sampling technique was used.

### 2.6. Data Collection Instrument

The instruments that were used for the data collection were the following validated assessment tools.

- Mental Health Service Satisfaction Scale (MHSSS), which was written in English and then translated and validated in Amharic (Cronbach's *α* = 0.92) [27]
- Oslo Social Support Scale (OSSS)—it is a three-item scale with Cronbach alpha of 0.75 and has a range value of 3-14, further categorized as follows: “poor support,” 3–8; “moderate support,” 9–11; and “strong support,” 12–14 [28]
- Clinical Decision-Making Involvement assessment tool—it is a tool that is used to assess the decision-making style of a physician on patient treatment with Cronbach alpha of 0.79, which has three parts [29]
- Clinical Global Impressions-Severity (CGI-S)—it is a seven-item scale with Cronbach alpha of 0.78, which is used to assess the level of clinical severity of psychiatric disorders on the basis of clinician experience in judging the level of illness like 0 = not assessed; 1 = normal, not at all ill; 2 = borderline mentally ill; 3 = mildly ill; 4 = moderately ill; 5 = markedly ill; 6 = severely ill; and 7 = extremely ill [30]
- The Alcohol, Smoking and Substance Involvement Screening Test (ASSIST-3.0) was adopted to assess the current status on alcohol, cigarettes, and khat and cannabis use of the participants. It was developed by WHO to detect psychoactive substance use and related problems in primary care patients with Cronbach alpha of 0.73 [31]

### 2.7. Data Collection Procedures

Face-to-face interview and document review were used to collect the data for this study. Four nurses who hold a bachelor degree and two psychiatry professionals were involved in data collection after they got two days of training about the objective of the study. After permission was obtained from respondents, the interviewers explained about the objective of the study and expectation from the respondents; then the questionnaires, which were designed to be conducted by the interviewers, were administered, and it took approximately 30 to 45 minutes to complete. Each data collector reviewed the card and recorded the card number of respondents who had completed the questionnaires, and in each day, the respondent's card number was shared to all data collectors to avoid redundancy of questionnaire. Each day, the principal investigator and the supervisors checked the completeness and quality of the collected data, incomplete questioners were excluded, and feedback was given to data collectors on a daily basis.

### 2.8. Study Variables

#### 2.8.1. Dependent Variable

Patient satisfaction was the dependent variable.

#### 2.8.2. Independent Variables

The independent variables are as follows:

- Sociodemographic-related factors: age, gender, educational status, marital status, place of residency, and income
- Psychosocial factors: social support
- Patient clinical characteristics: current substance use, current diagnosis of mental illness, duration of illness, comorbid medical illness, and clinical severity scale
- Service-related factors: decision-making style of clinician, waiting time, consultation time, family involvement in treatment, accessibility of service, and distance from the hospital

### 2.9. Data Analysis

The coded data were entered into EPI-DATA version 3.1 to minimize data entry error and then exported to SPSS version 22.00 for analysis. Descriptive statistics such as texts, percentage, graphs, and tables for categorical data and calculated mean and standard deviation for continuous variables were used. Simple linear regression was used to identify variables that are candidate for multiple linear regression at *P* < 0.25; and to adjust the confounder variables, multiple linear regression analysis was used; and variables at *P* < 0.05 determine the dependent variable independently. Before linear regression analysis was performed, assumptions of linear regression were checked such as normality was checked by using normal histogram curve and the Kolmogorov-Smirnov Test; linearity was checked by using (quantile–quantile) QQ plot and histogram; no outlier was found during outlier test; multicollinearity was checked by using variance inflation factor, and all variables had VIF < 2; homoscedasticity was checked by using Levene's test in which all variables that were *P* > 0.05 indicate no heteroskedasticity. Independent observation was checked by Durbin-Watson value, and the value of this finding was 1.95.

### 2.10. Data Quality Assurance

The possible maximum sample size with nonresponse rate was calculated. Standard and carefully designed questionnaires were used and translated to local languages Afaan Oromo and Amharic by two different persons and back-translated to English. Pretest was done among 5% of the participants in Shenen Gibe Hospital who attend their treatment at an outpatient service to check for the understandability, reliability, and clarity of the questionnaire before the actual data collection. The internal consistency of service satisfaction measurement items in pretest was Cronbach′s alpha = 0.814.

Data collectors were not wearing hospital gown to avoid the reluctance of patients to give reliable information.

## 3. Result

### 3.1. Sociodemographic Characteristic of Respondents

Complete data were obtained from 414 respondents with 98% response rate. Among the total respondents, 286 (69.1%) were males, and the mean ± SD age of respondents was 33 ± 9, which ranged from 18 to 67 years, and the majority of the 254 participants (61.4%) were Muslims. More than half of the respondents, 214 (51.4%), were single, followed by married, 163 (39.4%). Nearly one-third of the respondents, 137 (33.1%), attended their education up to primary school [1–8]; one-fourth of the respondents, 88 (21.3%), were government employees. The median income of the respondents was 1000 with interquartile range of 500 ETB. The majority of the respondents, 316 (76.3%), were from an urban area; the median distance of respondents from the hospital was 35 (Min = 1, Max = 300) km; and the majority of the respondents, 401 (96.9%), had health insurance (Table 1).

### 3.2. Clinical-Related Factor of Respondents

The mean ± SD of age onset of the illness of the respondents was 27 (±7) years, which ranges from 15 to 61 years; and the mean total duration of illness was five with (SD ± 4), which ranges from 1 to 25 years. The mean ± SD of waiting time of respondents was 56 (±25) minutes, which ranges from 10 to 120 minutes; and consultation time was 14 (±5) minutes, which ranges from 5 to 40 minutes. Nearly half of the respondents had a history of admission, 216 (52.2%), prior to data collection period.

Most of the respondents, 269 (65%), respond as they were attending modern treatment for the first time; and 145 (35%) of respondents were attending traditional treatment at for the first time. Out of them, almost all, 130 (89.6%), used religious treatment (holy water and prayers). 20 (4.6%) of the respondents had comorbid medical illness, and 242 (58.2%) of the respondents were not current substance users (Table 2).

**Figure 1:**
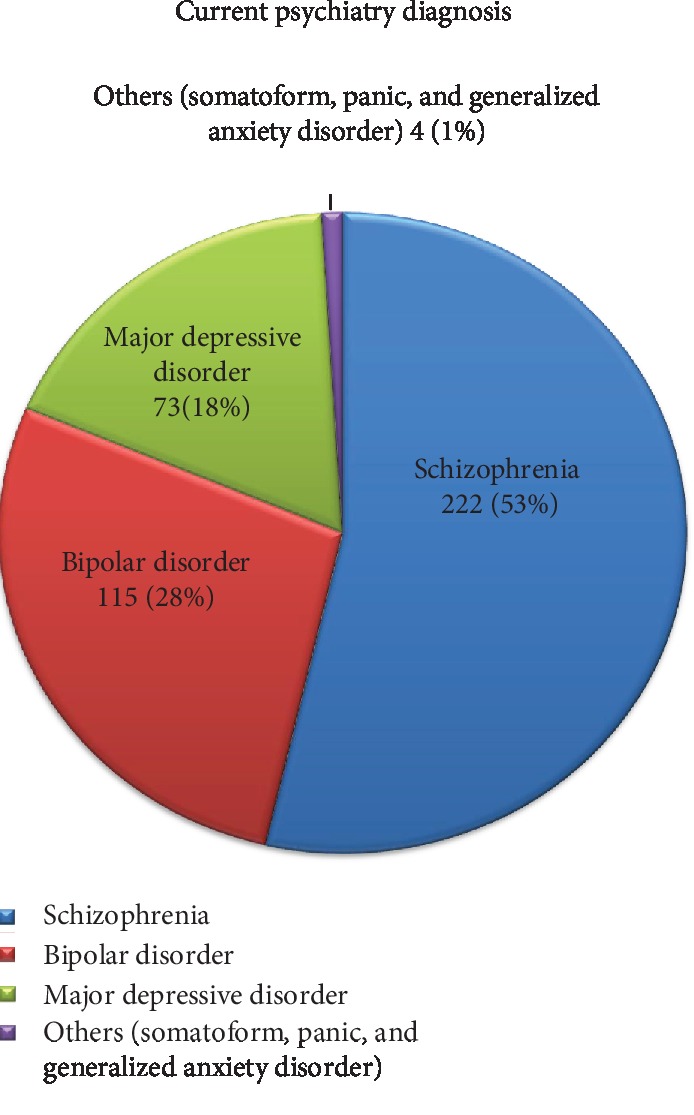
Type of current psychiatry diagnosis of respondents who attend their treatment at Jimma University Medical Center, outpatient psychiatry clinic, southwest Ethiopia, 2019 (*N* = 414).

Of all respondents, more than half of them, 222 (53%), had a diagnosis of schizophrenia (Figure 1).

**Figure 2:**
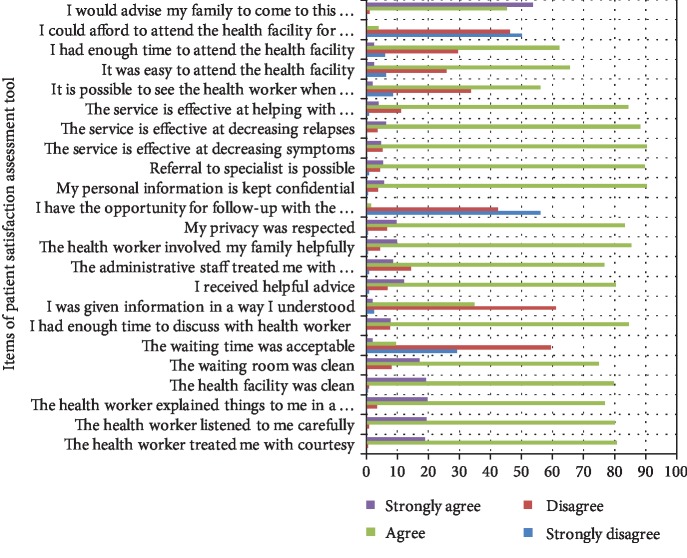
Patient response for each mental health service satisfaction assessment item at Jimma University Medical Center, southwest Ethiopia, 2019 (*n* = 414).

From all items used to measure patient satisfaction, more than two-thirds of respondents responded that they disagree and strongly disagree for the items the opportunity to be followed up by the same health worker, affordability of service, and acceptability of waiting time; but for the rest of the items, the respondents responded that they agree and strongly agree (Figure 2).

### 3.3. Magnitude of Patient Satisfaction

The mean score of patient satisfaction among respondents who attend their treatment at Jimma University Medical Center, outpatient psychiatry clinic, was 71/92 (95% CI (70.8-71.1)), in which 53% of them score above the mean patient satisfaction score when it was transformed into percentage score of (actual − minimum/maximum − minimum)∗100 = 50.3%.

From all items used to measure patient satisfaction, most respondents—366 (88.6%), 406 (98.3%), and 397 (96.1%)—responded disagree and strongly disagree for the items the waiting time was unacceptable, lack of opportunity for follow-up by the same health worker, and could not afford to attend the health facility for treatment, respectively; but for the rest of the items, they responded agree and strongly agree.

### 3.4. Factors Associated with Patient Satisfaction

After adjustment of potential confounders by using multiple linear regression with *P* value < 0.05 (stepwise method of analysis), sex, educational status, residence, current psychiatry diagnosis, distance from the home, substance use, waiting time, and social support independently predicted patient satisfaction score of the patient. Being male (*β* = −0.651, 95% CI (-0.969, -0.332), *P* = 0.001), having secondary and above educational status (*β* = −1.250, 95% CI (-1.765, -0.735), *P* = 0.000), living in a rural area (*β* = −1.358, 95% CI (-1.687, -1.030), *P* = 0.000), having a diagnosis of major depressive disorder (*β* = 1.719, 95% CI (1.332, 2.106), *P* = 0.000) and bipolar disorder (*β* = 1.203, 95% CI (0.890, 1.516), *P* = 0.000), increase in distance from the hospital (*β* = −3.250, 95% CI (-4.662, -2.450), *P* = 0.000), having a history of current substance use (*β* = −1.719, 95% CI (-2.015, -1.423), *P* = 0.000), increase in waiting time (*β* = −3.853, 95% CI (-4.701, -2.205), *P* = 0.000), and strong social support (*β* = 0.456, 95% CI (0.231, 0.654), *P* = 0.001) were variables significantly associated with patient satisfaction (Table 3).

## 4. Discussion

This study found that the overall percentage of patient satisfaction was 50.3% (95% CI (48.4%–51.2%)) with mean patient satisfaction score of 71/92 (CI = 70.8-71.17).

This study finding showed that the overall percentage score of patient satisfaction was 50.3% (95% CI (48.4%–51.2%)), and this was the same with the study done in Nigeria (45%) (95% CI (0.34-0.56)) [15] and Addis Ababa (57%) (95% CI (0.46-0.68)) [18] but lower than the score of the studies done in Ireland (90.7%) (95% CI (0.81-0.99)) [14], Pakistan (92.7%) (95% CI (0.86-0.98)) [32], India (87.28%) (95% CI (0.82-0.92)) [33], and South Africa (72.9%) (95% CI (0.56-0.88)) [16]; and this difference might be due to the difference in the number of sample size, type of measurement tool used, socio-demographic characterstics study participant, and differences in mental health literacy, mental health service, and availability of alternative mental health service within the country. In addition, this study finding was lower than that of the studies done in Ethiopia, Mekelle (72%) (95% CI (0.64-0.80)) [34] and Dessie (61.2%) (95% CI (56.72-65.68)) [35]; this difference might be due to the difference in sample size and assessment tool wherein they used CSQ and CPOSS, respectively, and also, this finding is lower than that of the studies done in Gondar (77%) (95% CI (0.70-0.84)) [17], which might be explained by the difference in sample size and study participants because in this study, most of the respondents were male, which is associated with lower patient satisfaction score as evidenced by this study result.

This study showed that 372 (90%) of respondents respond agree and strongly agree to the item received helpful advice from professionals, and this result was similar with that of the study done in Gondar [17] in which 92.6% of respondents said good, very good, and excellent to the item, and this similar result might be because of a clinician's similar responsibility to advise their patients in the clinic.

This study found that 385 (93%) of respondents responded strongly disagree and disagree to the item being followed up by the same health professional during follow-up visit, and this finding was two times higher than that of the study done in Gondar [17] in which 40% of respondents said poor and fair to the same item; this difference might be due to the variation in sample size, work setting, and staff profile and due to different health care providers during various visits, which can confuse a patient in who to contact during need for help; moreover, the majority of the patients are also unwilling to closely approach their health care provider and tell details about their life. This might also be very important to ensure an appropriate diagnosis and follow-up.

This study found that 405 (98%) of respondents agree and strongly agree to the item cleanliness of the waiting area, which was similar with the study done in Gondar [17], and showed that the majority (92%) of participants were satisfied regarding the location and cleanliness of the outpatient care area.

This study found that being male decreases the patient satisfaction score by 0.65 units. Being male (*β* = −0.651, 95% CI (-0.969, -0.332), *P* = 0.001) was a similar result with the study done in Dessie [35] and Nigeria [15] (adjusted odds ratio (AOR) = 0.51, 95% CI: 0.37, 0.96), and this might be because males show poor adherence to treatment and higher use of psychoactive substance than that do females, which make them less responsive to psychiatric treatment, which was evidenced by the study done in Zurich [36].

This study found that living in a rural area, as compared with an urban area, decreases the patient satisfaction score by 1.35 units (*β* = −1.358, 95% CI (-1.687, -1.030), *P* = 0.000), which was similar with study done in Addis Ababa [18]; and this might be because those respondents who came from a rural area mostly lived far and have problem in transportation and access to medication, their chance to be visited by health professionals was less likely as compared with the chance of those from urban area residences.

This study found that having secondary and above level education decreases patient satisfaction score by 1.25 units (*β* = −1.250, 95% CI (-1.765, -0.735), *P* = 0.000), which was a similar result with the study done in Nigeria [37] and Mekelle [34]; and this similar result might be because those respondents who had higher education had high expectations of service.

This study found that having a diagnosis of major depressive disorder increases the patient satisfaction score by 1.71 units (*β* = 1.719, 95% CI (1.332, 2.106), *P* = 0.000) and bipolar disorder by 1.20 units (*β* = 1.203, 95% CI (0.890, 1.516), *P* = 0.000), as compared with schizophrenia, which was supported by the studies done in Canada [36], India [38], Dessie [35], Mekelle [34], and Gondar [17]; this similar result might be because psychotic disorders, as compared with other types of mental illness, are debilitating, which especially affects patients' level of understanding of their external world.

This study found that having current substance use history decreases the patient satisfaction score by 1.71 units than not having current substance use (*β* = −1.719, 95% CI (-2.015, -1.423), *P* = 0.000), which was supported by the two different studies done in the USA [39] and Los Angeles [40], which might be because substance use affects the normal therapeutic effect of medication, which then leads to poor adherence, more relapse, and poor outcome and functionality, which on the whole affects the satisfaction score of the patient.

This study found that as the waiting time increases, the patient satisfaction score decreases by 3.85 units (*β* = −3.853, 95% CI (-4.701, -2.205), *P* = 0.000), which was a similar result finding with the studies done in Bangladesh [41], Wolaita Sodo [42], and Mekelle (AOR = 0.01; 95% CI: 0.002, 0.07) [34], and this similar result might be because as waiting time of patients increases, their less chance to talk with the therapist about their problem leads to poor therapeutic alliance; moreover, for those respondents who live far and in rural areas, their interset to be interviewed timely as soon as they arrived at the hospital to return back to home early were not addressed, which all results in negative attitude of patients towards the service given by the hospital.

This study found that having strong social support score increases patient satisfaction score by 0.456 units (*β* = 0.456, 95% CI (0.231, 0.654), *P* = 0.001), which was a similar result with the study done in Egypt and Ghana [43, 44], and this similar result might be because good social support helps the patients in accessing service through different means, for example, accompanying the patients to go to the hospital, buying medication as needed, reminding the patients to take their medication on time, and also providing emotional support at home, which facilitate treatment outcome and functionality of patients, which all result in positive attitude of patients towards service.

This study found that as the distance of home from the hospital increases, the patient satisfaction score decreases by 3.25 units (*β* = −3.250, 95% CI (-4.662, -2.450), *P* = 0.000), which was a similar result with the study done in Dessie [35] and Addis Ababa (AOR = 3.21, 95% CI: 2.0, 7.52) [18], which might be because living far affects timely accessibility of service including attending in time for follow-up, buying medication as needed, and consulting with mental health professionals.

## 5. Conclusion

This study found that only half of respondents have a score above the mean of patient satisfaction score and that most of the respondents responded strongly disagree to the items acceptability of waiting time, opportunity to be followed up by the same professional, and affordability of treatment. Being male, living in a rural area, having secondary and above level of educational status, having schizophrenia, increase in distance of home from the hospital, increase in waiting time, current substance use history, and having low social support score were inversely correlated with patient satisfaction score. So working on the identified modifiable factors with respected stakeholders, which hinder patient satisfaction at outpatient psychiatry service, will be the solution to increase satisfaction of patients, improve the outcome of patient, and achieve quality of service.

### 5.1. Recommendation

It is better to have continuous and periodic supervision of health institutions for timely feedback and intervention of factors affecting patient satisfaction.

## Figures and Tables

**Table 1:** Sociodemographic characteristics of respondents at Jimma University Medical Center, southwest Ethiopia, 2019 (*n* = 414).

Variable	Category	Frequency (*N* = 414)	Percentage (%)
Sex	Male	286	69.1
	Female	128	30.9
Religion	Muslim	254	61.3
	Orthodox	107	25.8
	Protestant	50	12.1
	Others^∗^	3	0.71
Marital status	Single	214	51.7
	Married	163	39.4
	Divorced	31	7.5
	Others^∗∗^	6	1.4
Educational status	No education	33	7.9
	Primary	137	33.1
	Secondary	126	30.4
	More than secondary	118	28.5
Occupation	Student	29	7.0
	Housewife	39	9.4
	Merchant	56	13.5
	Government employee	88	21.3
	Farmer	74	17.9
	Private work	87	21.0
	Jobless	36	8.7
	Others^∗∗∗^	5	1.2
Residency	Urban	316	76.3
	Rural	98	23.7
Health insurance	Yes	401	96.9
	No	13	3.1

Others: ^∗^Jehovah, Catholic; ^∗∗^widowed; and ^∗∗∗^pension.

**Table 2:** Distribution of clinical- and service-related factors of respondents in Jimma University Medical Center, outpatient psychiatry clinic, southwest Ethiopia, 2019 (*N* = 414).

Variable	Category	Frequency	Percentage
Having comorbid medical illness	Yes	19	4.6
	No	395	95.4
Severity of the illness	Normal, not at all	49	11.8
	Borderline mentally ill	316	76.3
	Mildly ill	49	11.8
Social support scale	Poor	240	58.0
	Moderate	149	36.0
	Strong	25	6.0
Current substance use history	Yes	172	41.5
	No	242	58.2
Clinical decision style of respondents	Passive decision	402	97.1
	Others^∗^	12	2.9
History of admission	Yes	216	52.2
	No	198	47.8
First trail treatment	Modern	269	65.0
	Traditional	145	35.0

Others: ^∗^active and shared decision.

**Table 3:** Linear regression analysis of respondents who attend their treatment at Jimma University Medical Center, outpatient psychiatry clinic, southwest Ethiopia, Jimma, 2019 (*n* = 414).

		Simple linear regression		Multiple linear regression			
Variables	Category	Unstandardized coefficients		Unstandardized coefficients		95% CI	
		*B*	Sig	*B*	Sig	Lower	Upper
Sex	Male	-0.751	0.000^∗^	-0.651	0.000^∗∗∗^	-0.969	-0.332
	Female			1			
Age	Age	0.005	0.529				
Religion	Religion	0.126	0.286				
	Muslim	-0.043	0.809				
	Protestant	0.360	0.176^∗^	0.240	0.268	-0.186	0.666
	Catholic	1.000	0.521				
Marital status	Married	-0.174	0.278				
	Divorced	-0.827	0.005^∗^	-0.698	0.070	-1.229	-0.167
	Widowed	0.017	0.979				
Educational status	No formal education			1			
	Primary	-0.692	0.007^∗^	-0.682	0.007^∗^	-1.172	-0.192
	Secondary	-0.649	0.013^∗^	-0.629	0.013^∗^	-1.125	-0.132
	More than secondary	-1.34	0.000^∗^	-1.250	0.000^∗∗∗^	-1.765	-0.735
Occupational status	Merchant	-0.576	0.072^∗^	-0.476	0.453	-0.271	-0.722
	Government employee	-0.228	0.438				
	Farmer	-0.470					
	Student	-0.727	0.121^∗^	0.786	0.217	-0.463	2.035
	Private work	-0.365	0.053^∗^	-1.175	0.081	-0.145	-2.495
	Jobless	-0.530	0.216^∗^	0.324	0.610	-0.926	1.575
	Pension	-0.508	0.134^∗^	0.230	0.731	-1.085	1.545
Monthly income	Monthly income	-0.001	0.485				
Residence	Urban			1			
	Rural	-1.558	0.000^∗^	-1.358	0.000^∗∗∗^	-1.687	-1.030
Distance in km	Distance from hospital	-3.31	0.000^∗^	-3.250	0.000^∗∗∗^	-4.662	-2.450
Insurance status	No free insurance	-0.020	0.964				
Onset of psychiatry illness	Age at first onset of illness	-0.014	0.170^∗^	-0.009	0.259	-0.025	0.007
Duration psychiatry illness	Total duration of illness	-0.089	0.140^∗^	-0.052	0.161	-0.081	-0.024
Admission status	No admission history	0.492	0.041^∗^	0.343	0.071	0.080	0.605
First contact of treatment	Traditional treatment	-0.093	0.559				
Comorbid illness	No current comorbid medical illness	1.454	0.030^∗^	0.864	0.064	0.285	1.442
Current substance use	Yes	-2.319	0.000^∗^	-1.719	0.000^∗∗∗^	-2.015	-1.423
	No			1			
Waiting time in minutes	Waiting time	-4.34	0.000^∗^	-3.853	0.000^∗^	-4.701	-2.205
Consultation time	Consultation time	0.001	0.908				
Social support	Low social support			1			
	Moderate social support	0.315	0.003^∗^	0.272	0.002^∗^	0.231	0.654
	Strong social support	0.567	0.002^∗^	0.456	0.001^∗^	0.412	0.876
Current psychiatry diagnosis	Bipolar disorder	1.245	0.000^∗^	1.203	0.000^∗∗∗^	0.890	1.516
	Major depressive disorder	1.761	0.000^∗^	1.719	0.000^∗∗∗^	1.332	2.106
	Schizophrenia			1			
Severity of illness	Borderline mentally ill	0.056	0.815				
	Mildly ill	-0.367	0.241^∗^	-0.005	0.979	-0.391	-0.002
Decision-making style of clinician	Shared decision	-0.457	0.615				
	Passive decision	-0.590	0.399				

^∗^Significant at *P* value < 0.25, during simple linear regression, selected for multiple linear regression (1 = reference, ^∗∗∗^*P* < 0.001, ^∗∗^*P* < 0.01, ^∗^*P* < 0.05, stepwise analysis, adjusted *R*^2^ = 0.668%).

## Data Availability

The materials and data of this study are available from the corresponding author upon request.

## Abbreviations

CDIS-P: Clinical Decision-Making Involvement and Satisfaction—Service User
CGI-S: Clinical Global Impression-Severity scale
ICCMH: Integrated Clinical and Community Mental Health
IRB: Ethical review board
JUMC: Jimma University Medical Center
LMICs: Low- and middle-income countries
MARS: Medication adherence rating scale
MHSSS: Mental Health Service Satisfaction Scale
OPD: Outpatient department
OSSS: Oslo Social Support Scale
*P* value: Probability value
PWMI: People with mental illness
SPSS: Statistical Package for the Social Sciences
SWLS: Satisfaction with Life Scale
WHO: World Health Organization.

## Additional Points

*Declarations*. We declare that this research paper is an original work and has not been submitted to any journal before.
